# It's the deceiver, not the receiver: No individual differences when detecting deception in a foreign and a native language

**DOI:** 10.1371/journal.pone.0196384

**Published:** 2018-05-03

**Authors:** Marvin K. H. Law, Simon A. Jackson, Eugene Aidman, Mattis Geiger, Sally Olderbak, Sabina Kleitman

**Affiliations:** 1 School of Psychology, University of Sydney, Sydney, Australia; 2 Institute for Psychology and Education, Ulm University, Ulm, Germany; Leiden University, NETHERLANDS

## Abstract

Individual differences in lie detection remain poorly understood. Bond and DePaulo’s meta-analysis examined judges (receivers) who were ascertaining lies from truths and senders (deceiver) who told these lies and truths. Bond and DePaulo found that the accuracy of detecting deception depended more on the characteristics of senders rather than the judges’ ability to detect lies/truths. However, for many studies in this meta-analysis, judges could hear and understand senders. This made language comprehension a potential confound. This paper presents the results of two studies. Extending previous work, in Study 1, we removed language comprehension as a potential confound by having English-speakers (N = 126, mean age = 19.86) judge the veracity of German speakers (n = 12) in a lie detection task. The twelve lie-detection stimuli included emotional and non-emotional content, and were presented in three modalities–audio only, video only, and audio and video together. The intelligence (General, Auditory, Emotional) and personality (Dark Triads and Big 6) of participants was also assessed. In Study 2, a native German-speaking sample (N = 117, mean age = 29.10) were also tested on a similar lie detection task to provide a control condition. Despite significantly extending research design and the selection of constructs employed to capture individual differences, both studies replicated Bond and DePaulo’s findings. The results of Study1 indicated that removing language comprehension did not amplify individual differences in judge’s ability to ascertain lies from truths. Study 2 replicated these results confirming a lack of individual differences in judge’s ability to detect lies. The results of both studies suggest that Sender (deceiver) characteristics exerted a stronger influence on the outcomes of lie detection than the judge’s attributes.

## 1. Introduction

The ability to detect when others are attempting to deceive us is important, but empirical research suggests that we are poor at lie detection. For example, a common finding in lab-based studies is that, on average, people perform no better than chance when judging whether another is lying or telling the truth [1, 2]. Similar to cognitive abilities (intelligence) or personality traits, it has been suggested that the ability to detect deception varies between individuals [3, 4]. That is, some individuals may be capable of detecting deception better than others despite this ability appearing to be negligible at an aggregate level. Individuals who supposedly possess the greatest skill in detecting deception have been referred to as lie detection ‘wizards’ [5]. Some evidence for such individual differences exists. For example, members of the US secret service, a handful of law enforcement agents, and clinical psychologists have been found to perform above chance when detecting lies [6, 7]. Some authors, however, suggest that statistical or methodological artefacts can explain these results [8].

It has been demonstrated that the “cognitive load approach” as a sole explanatory principle is insufficient for lie detection [9]. This has led to a renewed focus on para-verbal and non-verbal cues, and individual's capacity to detect them. The latest integrated model [9] combines newer versions of Baddeley’s [10, 11] working memory model with constructs of mental control [12, 13]. It enables verbal content cues, nonverbal, para-verbal, and linguistic cues to be investigated within a single framework. This framework emphasises the importance of individual differences in language ability and working memory capacity as well as arousal, stress and emotion [14]. Our study examines inter-individual variability of individuals' capacity to discriminate between lies and truths when language comprehension is removed from the equation. This paper presents results of two studies. In Study 1, a verbal message is presented in a foreign language using a cross-language deception technique (see below). In Study 2, verbal messages are presented to native German speakers to control for language comprehension.

### 1.1. The lie detection paradigm

This research, albeit extending it in several important ways, employed a typical deception detection paradigm in which participants (“judges”) had to determine whether people in video and audio recordings (“senders”) are lying or telling the truth. To investigate individual differences in the ability to detect deception, researchers typically focus on judge ability: the percentage of senders correctly judged to be telling the truth or lying. The chance level is around 50%. Other variables of interest can also be calculated (summarised in Table 1). *Judge credulity* is measured by the percentage of “Truth” decisions (regardless of their accuracy), and it reflects the tendency of the “*judge”* to trust others. *Sender detectability* and *credibility* refer to information about *the people who are being judged* as telling the truth or lying. For each *sender*, their *detectability* is measured as the percentage of judges who make a correct decision about them, and their *credibility* is measured by the percentage of judges who decide that they are telling the truth (regardless of accuracy).

**Table 1:** Dependant variables investigated in Bond and DePaulo [15].

Variable Name	Description	Method of Calculation
*Judge ability*	The capacity to accurately determine whether others are lying or telling the truth	Percentage of senders correctly judged as telling the truth or lying
*Judge credulity*	The extent in which an individual judges others to be trustworthy	Percentage of senders judged as telling the truth (regardless of accuracy)
*Sender detectability*	The extent in which an individual can be accurately detected when lying or telling the truth	Percentage of judges who correctly decide that the sender is telling the truth or lying
*Sender credibility*	The extent an individual is trusted by others	Percentage of judges who decide that the sender is telling the truth (regardless of accuracy)

### 1.2. Individual differences in deception detection

Extensive research on these variables suggests that individual differences in the ability to discriminate truth-telling from lying (judge ability) are negligible. Bond and DePaulo [15, 16] best addressed this question in a meta-analysis of 247 samples, including 12704 judges (participants) and 6060 senders (items). Within their meta-analytic model, Bond and DePaulo were able to separate within-study measurement errors from meaningful variance in judge ability by examining patterns of results across studies. Within their findings, three measurement-corrected methods for evaluating the presence of meaningful individual differences in deception detection were empirically reported. One, reasonable internal consistency for judge ability following a ‘truth’ or ‘lie’ response [15, 16, 17] (see Equation A in S1 Appendix). Two, comparable or larger variability in judge ability than judge credulity, sender detectability and sender credibility (see Equation B in S1 Appendix). Three, positive and significant correlations between judge ability when calculated separately for senders who were telling the truth and senders who were lying. Overall, there was null evidence for each of these three methods. The estimated coefficient Alpha for judge ability was .13, and Spearman-Brown coefficient was .22, which are considerably lower than acceptable levels of internal consistency for research (around .70, [18]). Similar estimates for the remaining variables were higher (for judge credulity, sender detectability and sender credibility, the estimated coefficient alpha was .43, .67 and .75 and Spearman-Brown coefficient was .75, .70 and .81 respectively). Examining the standard deviations, variability in judge ability was considerably lower than variability in the other three variables. Finally, the correlation of judge ability computed separately for truth-telling and lying senders was close to zero and negative (*r* = -.09). These results emerged across all studies despite differences in deception modality (sender presented with audio and video, audio only, video only), sender motivation (getting away with lying would opt a prize), the interaction between sender and judge (e.g. whether the two interacted during the deception), sender preparedness (how much time for preparation of the lie was given), experience of judge as well as baseline exposure (how the sender would act when not lying) (replicated in [19]’s meta-analysis). The overall conclusion made in Bond and DePaulo [15, 16] was that decisions made on deception detection tasks are primarily driven by factors related to judge credulity, and sender attributes influencing detectability and credibility. Thus, if individual differences in deception detection do exist, we are likely able to observe them only when the judges’ access to the information about the sender is limited or removed. This belief has also been supported by [20], who looked at the effects of sender demeanour (appearance of honesty) on deception detection. This study found that honest-looking truth-tellers and deceptive-looking liars (demeanour-veracity-matched) were detected more accurately than senders whose appearance did not match their actual honesty (demeanour-veracity-mismatched). Our research therefore sought to examine the hypotheses postulated by Bond and DePaulo [15, 16] about individual differences in deception detection when access to a critical source of sender information is removed (Study 1) and when it is present (Study 2).

### 1.3. Language comprehension

The source of sender information to be removed in this research is language comprehension, specifically the semantics. In lie detection tests, the sender can be seen, heard, or seen and heard to be speaking a language that judges can comprehend [21]. Although audio-only deception paradigms produce detection rates that are similar to those observed under the combined audio/video deception presentation, language is considered to be a crucial source of information for detecting deception. To demonstrate, deception can be inferred when a sender makes a false statement about known information. In applied settings, lie detection methods–including those employed within the judicial system and other organisations–often depend on a content-based analysis of the words being uttered and the meaning that surrounds them [22, 23]. The use of such content-based analyses has been shown to increase the accuracy of lie detection to 71% [24], significantly above chance level. This is particularly prominent within forensic contexts, where the use of content-based strategies has been shown to increase deception detection [25]. In particular, strategic use of evidence in interrogation (e.g. late evidence disclosure) has been shown to increase discriminatory content-based cues between liars and truth-tellers [26]. Although strategy use is important, most individuals in the general population would not have been exposed to such training. Moreover, there might be a potentially misleading aspect of language for *untrained* samples. That is, when the general population is sampled, aphasic patients, whose language processing abilities are impaired, perform significantly better at detecting deception than controls who perform no better than chance [27]. Thus, in our current research, an alternative approach was taken to examine whether the use of non-verbal cues might enhance lie detection when a population of 1st year psychology students is used. In Study 1, we asked English-speaking judges to complete a typical lie-detection task, with the exception being that the senders were speaking a foreign language (German). By having cross-language deception with judges being unable to comprehend the senders’ content, individual differences in lie detection were expected to emerge.

### 1.4. Additional individual differences variables

The evidence in favour of individual differences being involved in lie detection were assessed via the three measurement-correct methods described above [15, 16], as well as an additional method. The additional method is based on the assumption that should individual differences in deception detection exist, then judge ability scores should correlate in a meaningful fashion with related constructs. Specifically, measures assessing cognitive abilities, personality, and emotional intelligence. Existing literature, however, indicates either weak or no relationship between lie detection accuracy and traditional measures of intelligence (either general or Gf/Gc) and personality variables [28, 29]. Auditory information like changes in pitch and pause durations [30] have been found to relate to lying [31]. No research, however, has examined the relationship between auditory abilities (as assessed by Auditory Processing or *Ga* measures) and lie detection. Our study aimed at bridging this gap and examining the role of *Ga* from the Cattell-Horn-Carroll (CHC) theory of Cognitive Abilities [30] in lie detection.

Theory suggests that people who have higher emotion perception, a branch of Emotional Intelligence (EI) defined as the ability to accurately perceive facial emotions, should be better in lie detection [32, 33]. However, the empirical support is limited. A study by Ekman, O’Sullivan, Friesen and Scherer [34] found that when coders applied the Facial Affect Coding System to code the facial expressions of liars, they were able to identify true and false emotional expressions, subsequently helping them to identify when targets were and were not lying. Hill and Craig [35] got similar results for the identification of true and false facial expressions of pain.

This paper consists of two studies. In Study 1, English-speaking judges completed a lie-detection task where the senders were speaking a foreign language (German; cross-language approach). We expect that removing language comprehension cues may enhance evidence in favour of individual differences in lie detection in general population. To control for language comprehension, in Study 2, German-speaking judgers completed the same lie-detection task (using a longer list of same and different senders).

Furthermore, by incorporating novel and conceptually relevant measures of auditory abilities and ability-based measures of emotional intelligence we aimed to extend existing literature, capturing more consistent evidence for the existence of individual differences in lie detection.

### 1.5. Aims and hypotheses

Overall, our aim is to examine whether individual differences in lie detection exist when the ability to comprehend the linguistic content of the liar or truth teller has been removed. To assess this, participants will complete a lie-detection test in which senders speak a foreign language. Participants will also complete traditional (*Gf* and *Gc*) and novel (*Ga*) measures of cognitive abilities, ability-based emotional intelligence (EI) and personality. A second control study with a German sample will also be examined, to replicate and extend Study 1, and also to compare as a baseline with the Australian sample. Evidence in favour of individual differences is expected to be demonstrated by:

1. Higher internal consistency for judge ability than those shown in Bond and DePaulo [16].
2. Increase in the variability of judge ability, and decrease in the variability of judge credulity, sender detectability and sender credibility when compared to Bond and DePaulo [15]
3. More positive and significant correlation of judge ability scores calculated separately for senders who were telling the truth and senders who were lying than in Bond and DePaulo [15]
4. Assuming there is evidence for individual differences in judge ability, we expect significant correlations of judge ability with measures of cognitive abilities (especially *Ga*), and ability-based measures of emotional intelligence.

## 2. Study 1

### 2.1. Methods

#### 2.1.1. Participants

The current study consisted of 126 first-year psychology undergraduates (92 female, *M*_age_ = 19.86, *SD* = 3.53, age range: 17–52). Data for the lie detection task was removed for three participants who indicated that they understood German fluently. Results for three participants in the Talk Masking task and six participants in the BEFKI-Gf task were removed due to data being missing or responses occurring outside the allowed range (evidence that the task instructions had been misunderstood). Additionally, results for the tests administered in the second portion of the study were not available for one participant. The final sample size was 123 for analyses relating to the lie detection paradigm. Missing values were not imputed and treated as pairwise for analyses reported. Ethics approval was obtained at the University of Sydney with Project Number: 2015/229.

#### 2.1.2. Measures

Table 2 summarises the measures used in this study. This study was part of a larger research protocol. Only measures relevant to this study were presented in this manuscript (full list is available from the corresponding author). Brief descriptions of the lie detection task are included below.

**Table 2:** Summary of measures used in this study.

Measures	Example Item	Reliability estimates from previous studies	Cronbach's alpha internal reliability from current study
*Emotional Intelligence*			
1. *Identification of Emotion Expressions from Composite Faces–Short Form* [36]. A measure of emotion perception using facial information. Each item consists of two halves of a face, a top and bottom half. Both halves of the face belong to the same person, however, each half displays a different emotion, with six total emotions used in the measure. Participants were asked to determine which emotion was shown in either the top or bottom half of the face, with six possible answers. Mean accuracy score was determined using [37]’s UHR to account for the false alarm rates of each emotion. To minimize testing time, only 36 of the 72 trials were administered.	See [36]	Full version: .81 [36]	.64
2. *Visual Search for Faces with Corresponding Emotion Expressions of Different Intensity* [36]. A measure of emotion perception with each item showing nine different facial expressions of the same person in a 3x3 grid. A majority of the faces displayed a single emotion and participants determined which of the faces displayed an emotion that differed from the majority. Overall scores were determined by calculating the difference between hit rates and false-alarm rates of the correct emotion within all items. To minimize testing time, only 20 of the 40 trials were administered.	See [36]	Full version: .86 [36]	.89
*Auditory Processing*			
1. *Talk Masking* [38]. A measure consisting of two voices being played at the same time. Participants identified the isolated words spoken by one voice, whilst a distractor voice continuously talked with increasing volume. Misspelt words were accepted as correct, but only if it was phonetically equivalent to a large degree to the correct word (e.g. relick to relic). To establish a range of isolated words, CELEX databases within the program N-Watch were used to determine both single syllable words, and common and uncommon two-syllable words [39, 40]. Accuracy scores were calculated by the percentage of correct responses with 24 isolated words as items.	See [38]	Split Half Reliability of .90 [38]	.77
2. *Tonal Memory* [38]. Participants complete pairs of tonal patterns, with each pair differing by one tone. Participants have to select which tone they thought differentiated the two patterns. Each pattern consisted of either 3 or 4 tones. Accuracy scores were calculated by the percentage of correct responses on three items (selected from 10 original items on the basis of psychometric analysis).	See [38]	Split Half Reliability of .91 [38]	.52
3. *Rhythm* [38]. Participants judged whether pairs of rhythmic patterns were equivalent or different. Each pair were either the same or different. Accuracy scores were calculated by the percentage of correct responses with ten items (selected from 20 original items on the basis of psychometric analysis).	See [38]	Split Half Reliability of .82 [38]	.64
*Personality*			
1. *Levenson Self-Report Psychopathy Scale* [41]. 26-item measure of psychopathic traits within the general population, with two separate factors of primary and secondary psychopathy. Participants rated the extent to which they agree with each statement on a scale from 1 (strongly disagree) to 4 (strongly agree). Scores for total psychopathy, primary psychopathy and secondary psychopathy were calculated by averaging item ratings for all items and items within each factor respectively.	Primary Psychopathy: 'I often admire a really clever scam' Secondary Psychopathy: 'Love is overrated'	Total Psychopathy: .86, Primary Psychopathy: .87 Secondary Psychopathy: .67 [42]	• Total: .82 • Primary: .85 • Secondary: .62
2. *Narcissism Personality Inventory-16* (NPI-16; [43]). Short form of the NPI-40. Measures narcissism, with each item consisting of pairs of sentences. Participants decided which sentence in each pair they felt most aligned with.	'I am an extraordinary person' and 'I am much like everybody else'	.72 [43]	.69
3. *Machiavellianism-IV* (Mach-IV; [44]). Scale measures the Machiavellianism personality trait, with participants rating the extent to which they agree with each item from 1 (strongly disagree) to 5 (strongly agree). Total scores were computed as the sum of all ratings. One item was removed due to a coding error.	'It is wise to flatter important people'	.82 [45]	.68
4. *Big 6 Personality Inventory* [46]. A 25-item measure of six personality traits; agreeableness, conscientiousness, extraversion, honesty/propriety, resiliency and originality/intellect. For each item, participants rated how strongly they agreed with the item from 1 (strongly disagree) to 5 (strongly agree), with overall mean personality item scores being calculated for each personality trait.	Agreeableness: 'I am inclined to forgive others' Conscientiousness: 'I like order' Extraversion: 'I laugh a lot' Honesty/Propriety: 'I would never take things that are not mine' Resilience: 'I rarely worry' Originality/Intellect: 'I am an extraordinary person'	.49-.76 [47]	• Agreeableness: .45 • Conscientiousness: .55 • Extraversion: .52 • Honesty/Humility: .50 • Resilience: .47 • Originality/Intellect .35
5. *Emotion-Specific Empathy* [48]. A measure of trait empathy, with participants rating the extent to which they agree with each item from -3 (disagree strongly) to 3 (agree strongly). There are twelve subscales which conform into two broader scales of affective and cognitive empathy. Mean ratings were calculated for both scales.	Affective Empathy: 'I easily feel sad when the people around me feel sad' Cognitive Empathy: 'It is easy for me to understand why others become scared when something frightening happens to them'	Twelve subscales: .76-.91 [48]	• Affective Empathy: .93 • Cognitive Empathy: .94
*Intelligence*			
1. *BEFKI-Gc* [49]. A subscale within the BEFKI test, measuring Gc. The test consisted of 32 general knowledge questions, with four possible answers for each question. Accuracy scores were calculated based on the percentage of correct responses.	'In which year did Columbus discover America?' 1642; 1492; 1502; 1367 [1492].	.88 [49]	.54
2. *Vocabulary Test* [50]. An 18 item measure of vocabulary, a sub-factor of crystallised intelligence. Participants decided which of five alternative answers most closely equates to the meaning of a target word. Accuracy scores were calculated based on the percentage of correct responses.	FEIGN: Pretend, Prefer, Wear, Be Cautious, Surrender [Pretend]	.67 to .81 [51, 52, 53]	.68
3. *BEFKI-Gf* [49]. a subscale within the BEFKI test, measuring fluid intelligence. The test provided 16 items, each of which consisted of a series of patterns. Participants then decided which two patterns continued the series. For each item, individuals would have to successfully determine both patterns before getting the item correct. Accuracy scores were calculated based on the percentage of correct responses.	See [49]	.74 [49]	.69
4. *Esoteric Analogies Test* (EAT; from the Gf/Gc Quickie Battery, [50]. A measure of both fluid and crystallised intelligence by using 24 verbal analogies as items. Participants selected which of four alternatives share the same relationship with a target word as the original pair. Accuracy scores were calculated based on the percentage of correct responses.	LOVE is to HATE as FRIEND is to: LOVER; PAL; OBEY; ENEMY [ENEMY]	.66 to .76 [51, 52, 54, 55]	.70

#### 2.1.3. Lie detection paradigm

In this test, participants judge whether each of twelve recordings of people speaking German (senders) are lying or telling the truth. Sender recordings include seven lies and five truths spoken. We employed unequal number of truth and lies videos to prevent the participants in this study from anticipating that a half of the stimuli should be lies. Overall ten senders were used to generate twelve videos–four females and six males. Two of the males appear in two recordings, one telling two truths and one telling one lie and one truth. Recordings are presented in one of three modalities: video only, audio only, and combined video and audio. For each modality, senders talked about an everyday topic (e.g., favourite music), their opinion about a controversial issue (e.g., legalisation of certain types of abortion), or their deviant behaviour (e.g., skipping school) which were then categorized into emotional (e.g., abortion) or a non-emotional topic (e.g., popular culture). Overall, participants judged two senders for each modality (3) x emotionality (2) format.

#### 2.1.4. Procedure

Participants completed the lie detection test, *Ga* tasks, Emotion Composite task, BEFKI-Gc, Vocabulary task and demographics in a supervised lab. All other measures were completed online in the participants’ own time.

### 2.2. Results

#### 2.2.1. Hypothesis 1

Table 3 presents descriptive statistics for variables from the lie detection test. Mean judge ability to detect lies for all items (total) was 45.4%: significantly below chance (*t*_122_ = -3.47, *p* < .001), and similar to prior research [21]. For each modality and emotionality condition, mean judge ability was either significantly below, or no different to, chance level. Replicating Bond and DePaulo [16]’s findings, reliability estimates were unacceptably low for judge characteristics and high for both sender detectability and credulity.

**Table 3:** Lie detection descriptives.

	Mean	SD	Min	Max	Coefficient α
Judge Ability					
Total	45.4	14.7	17.0	75.0	.15
Video and Audio	44.1	26.4	0.0	100.0	.29
Muted Video Only	46.5	21.3	0.0	100.0	-.24
Audio Only	45.5	27.5	0.0	100.0	.29
Emotional Context	52.0	18.1	17.0	100.0	-.17
Non-Emotional Context	38.8	19.3	0.0	83.0	-.00
Judge Credulity	49.0	12.3	16.7	75.0	-.25
Sender Detectability	45.4	16.3	18.7	69.9	.94
Sender Credulity	49.0	17.0	24.4	81.3	.94

Note: Scores of Lie Detection are calculated as percentages (%)

#### 2.2.2. Hypothesis 2

**Fig 1:**
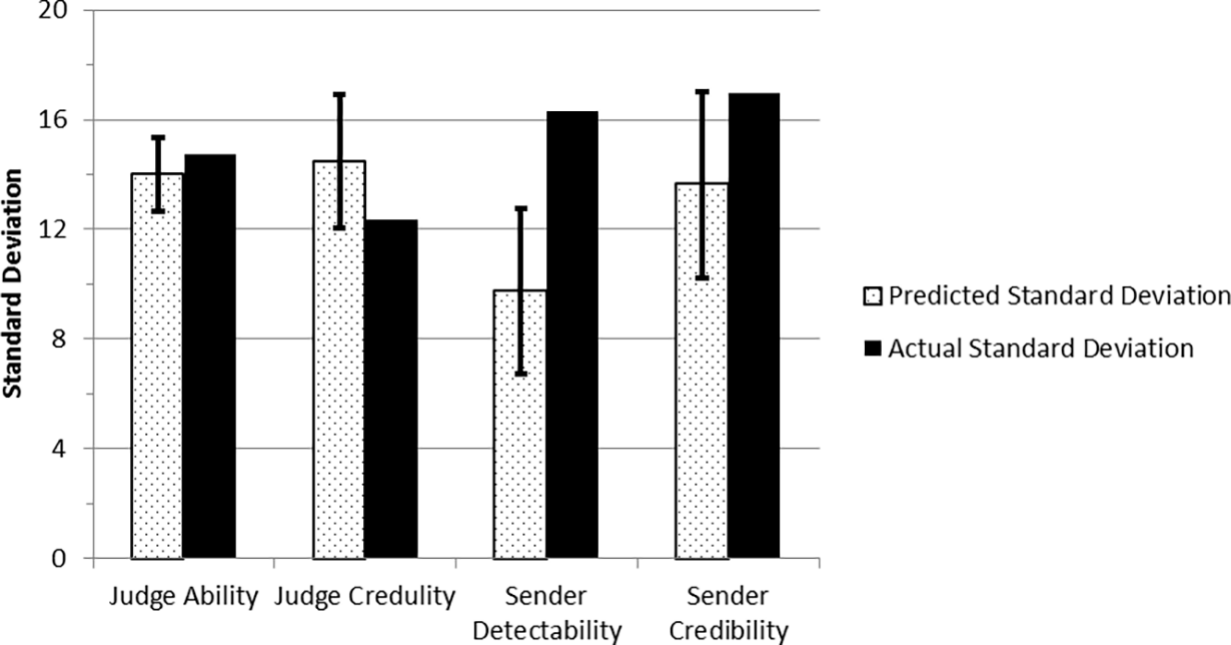
A comparison of the predicted standard deviation from Bond and DePaulo [15] with the actual standard deviation from the current experiment’s estimates of judge ability, judge credulity, sender detectability and sender credibility. Error bars represent the predicted standard deviations within a 95% Confidence Interval.

To compare variance of the judge and sender metrics in this study to prior research, we calculated the predicted standard deviations of these variables as recommended by Bond and DePaulo [15] using their meta-analysis data. That is, for each study included in their meta-analysis, standard deviations of the variables were calculated. These were each regressed on the inverse of the square root of n (number of judges or senders as applicable; see [15] for details) for each study. The resulting regression equations were used to calculate predicted standard deviations with confidence intervals for each variable given our sample size (123) and number of sender items (12). Fig 1 plots the predicted standard deviations, with confidence intervals, given Bond and DePaulo’s meta-analysis, beside the results from this study.

Bond and DePaulo’s meta-analytic results predicted a standard deviation of 14 for judge ability based on 12 senders. The standard deviation of judge ability in this Study was 14.72 (see Table 3), which is within the predicted 95% confidence interval margins. Following Bond and DePaulo’s analysis, we also compared the standard deviation of judges’ ability in determining truths against lies. The standard deviation of judge credulity also fell within the 95% confidence interval of the value predicted by Bond & DePaulo given 12 senders. The standard deviation of sender detectability fell above the 95% confidence interval of the value predicted by Bond & DePaulo (given 123 judges). However, similar to the judge variables, the standard deviation of sender credibility fell within the range predicted by Bond & DePaulo. That is, the standard deviation for three of the four variables examined fell within the range of observed values from prior research not controlling for language comprehension.

#### 2.2.3. Hypothesis 3

For each participant, judge ability was calculated separately for senders telling the truth or lying. While Bond & DePaulo found a small negative correlation between these separate scores, our data returned a small, marginally significant positive correlation (*r* = .18, *p* = .045).

#### 2.2.4. Hypothesis 4

Within the first three hypotheses, there was only some support for hypothesis 3. Thus, we did not expect any significant correlations between judge ability and other individual difference variables. The limited support for individual differences in judge ability would indicate that even if there were significant correlations, the estimates of judge ability are essentially noise. However, in order to complete our examination of potential individual differences in lie detection, we estimated correlations between judge ability and other individual difference variables. Performance on the Emotional Composite task correlated with total judge ability (*r* = -.19, *p* = .03) and judge ability calculated within the video and audio condition (*r* = -.25, *p* = .01). However, after controlling for false discovery rate using the Benjamini-Hochberg method [56] with PROC MULTTEST in SAS [57], neither correlation was statistically significant (*p*_*adj*_ = .97 and *p*_*adj*_ = .79 accordingly).

### 2.3. Discussion

Overall, Study 1 found little evidence to confirm individual differences in the ability to judge the veracity of verbal messages uttered in a foreign-language. The motivation for this work was to extend the findings of Bond and DePaulo [15, 16] by examining whether cross-language deception (removing language comprehension) would reveal evidence of individual differences in judge ability. Extending previous research, we also addressed all constructs suggested by Sporer’s integrated framework [9], including language ability (Gc), working memory (Gf), stress and emotion (EI), as well as abilities specific to detecting para-verbal cues (Ga). We then used a novel approach proposed by Bond and DePaulo [15, 16] consisting of four methods to test the presence of individual differences in judge ability. Judge ability as well as the other judge and sender characteristics were measured on a lie detection task where senders spoke a foreign language.

Firstly, while prior research has typically found judge ability to be at or just above chance levels, judge ability was below chance level accuracy in this study. This may be due to a demeanour-veracity-mismatch in senders as shown in [20].

Compared to prior research, a higher internal consistency for judge ability to detect lies in foreign-language spoken messages was expected in this study. However, this hypothesis was disconfirmed, with spearman-controlled internal consistency estimates for judge ability being so low as to suggest that these scores did not capture anything systematic about individuals’ ability to detect lies.

We expected greater variance accounted for by judge characteristics relative to sender characteristics. This hypothesis received no support with standard deviations of judge ability and credulity not differing significantly from the pattern of results found in previous research. The same result emerged for sender credibility, and only variance in sender detectability significantly increased compared to prior research. This last significant result may be due to larger variation in demeanour-veracity within the senders, with some senders appearing more mismatched and others appearing more matched [20]. The overall pattern of variance was the same as those found in [15]. Sender credibility accounted for the greatest amount of variance, followed by sender detectability, judge credulity and lastly judge ability. Even in the absence of language comprehension, the current results support Bond and DePaulo [15]’s conclusion that sender characteristics are most central for driving decisions on lie detection tests.

We also hypothesised that judge ability scores calculated separately for truth-telling and lying senders would correlate positively. Some support for this hypothesis was found in the form of a small but significant positive correlation observed between the separate judge ability scores. While this provides some support for individual differences in a lie-detecting ability, it should be noted that this correlation was weak (< .20). Thus, this result may be spurious and requires further replication.

Finally, we expected meaningful correlations to emerge between judge ability and theoretically related individual differences constructs. To address this, we assessed a large battery of tasks assessing a range of theoretically related constructs (both traditional and novel). The novel additions included two ability-based tests of emotional intelligence and three tests of auditory processing. After controlling for false discovery rate, no significant correlations between judges’ ability to detect lies and these theoretically related constructs were found. Our results therefore provide no support for the hypothesis that the removal of language comprehension might amplify individual differences in lie detection.

Next, we tested the same hypotheses from Study 1 using a native German-speaking sample, examining deception detection performance as well as standard deviations in judge ability, judge credulity, sender detectability, and sender credibility. One of the novel aspects of this study was to examine whether the use of nonverbal cues might enhance lie detection using a population whom are naïve to the language cues. Thus, it is important to compare the findings between two different populations, one that is completely naïve to the language cues and one that is not, as the explicit comparison between the two provides strong methodological grounds for our conclusions in relation to our hypotheses. Therefore, a second study has been conducted on a population of native German speakers using similar methodology and stimuli. It should be noted that combining the two populations in one common analysis is inappropriate due to two reasons. Firstly, Bond and DePaulo (2008a, b)’s methodology does not offer a way to control for the use of two different populations statistically. Secondly, having the analyses done on two different populations allows this research to be more transparent and easier to replicate—an important consideration given the current replicability crisis.

## 3. Study 2

### 3.1. Methods

#### 3.1.1. Participants

The sample consisted of 117 adults recruited for one of two studies from the German cities Ulm (*N* = 71) and Berlin (*N* = 46). The samples did not differ on age (*t*_(100)_ = .59, *p* = .56; 15 missed the answer) or gender ratio (*t*_(102)_ = -.26, *p* = .79; 13 missed the answer) or on their performance on the deception detection task (described below) and hence were combined. The total sample was primarily female (53%) and on average 29.10 years old (*SD* = 8.93, age range: 17–65). Ulm participants were recruited through advertisements posted online (e.g. on Facebook), on radio and face-to-face recruiting in the central city, while Berlin participants were recruited from eBay Minijobs and through paper flyers posted around the community. Participants individually consented to participate in the study and were financially compensated for participating. The study was conducted in accordance with the Declaration of Helsinki.

#### 3.1.2. Measures

Participants from Ulm were recruited for a study specifically assessing deception detection abilities. In addition to a demographics questionnaire, they completed 54 deception detection trials which presented videos from the same 10 stimulus persons used for the deception detection trials in Study 1. Participants from Berlin were recruited for a larger study on individual differences and completed a subset of 18 deception detection trials selected from the larger set administered to the Ulm participants. Although, the participants also completed several measures of personality and cognitive abilities (full list is available from the corresponding author), only measures relevant to this study were presented in this manuscript. Average performance across the 18 items presented in Ulm and Berlin did not significantly differ between the groups (*t*_(109.5)_ = 1.27, *p* = .21). Three of the 18 trials were also presented in Study 1.

### 3.2. Results

#### 3.2.1. Hypothesis 1

Table 4 presents descriptive statistics for variables from the lie detection test. Mean judge ability to detect lies for all items (total) was 55.7%: significantly above chance (*t*_116_ = 5.82, *p* < .01). Replicating Bond and DePaulo [16] and Study 1, reliability estimates were unacceptably low for judge characteristics and high for both sender detectability and credulity.

**Table 4:** Lie detection descriptive statistics for Study 2.

	Mean	SD	Min	Max	Coefficient α
Judge Ability	55.7	10.6	33.0	83.0	.03
Judge Credulity	55.0	12.2	22.0	78.0	.29
Sender Detectability	55.7	23.4	18.0	89.9	.97
Sender Credibility	55.0	23.5	11.0	87.0	.97

Note: Scores of Lie Detection are calculated as percentages (%)

#### 3.2.2. Hypothesis 2

**Fig 2:**
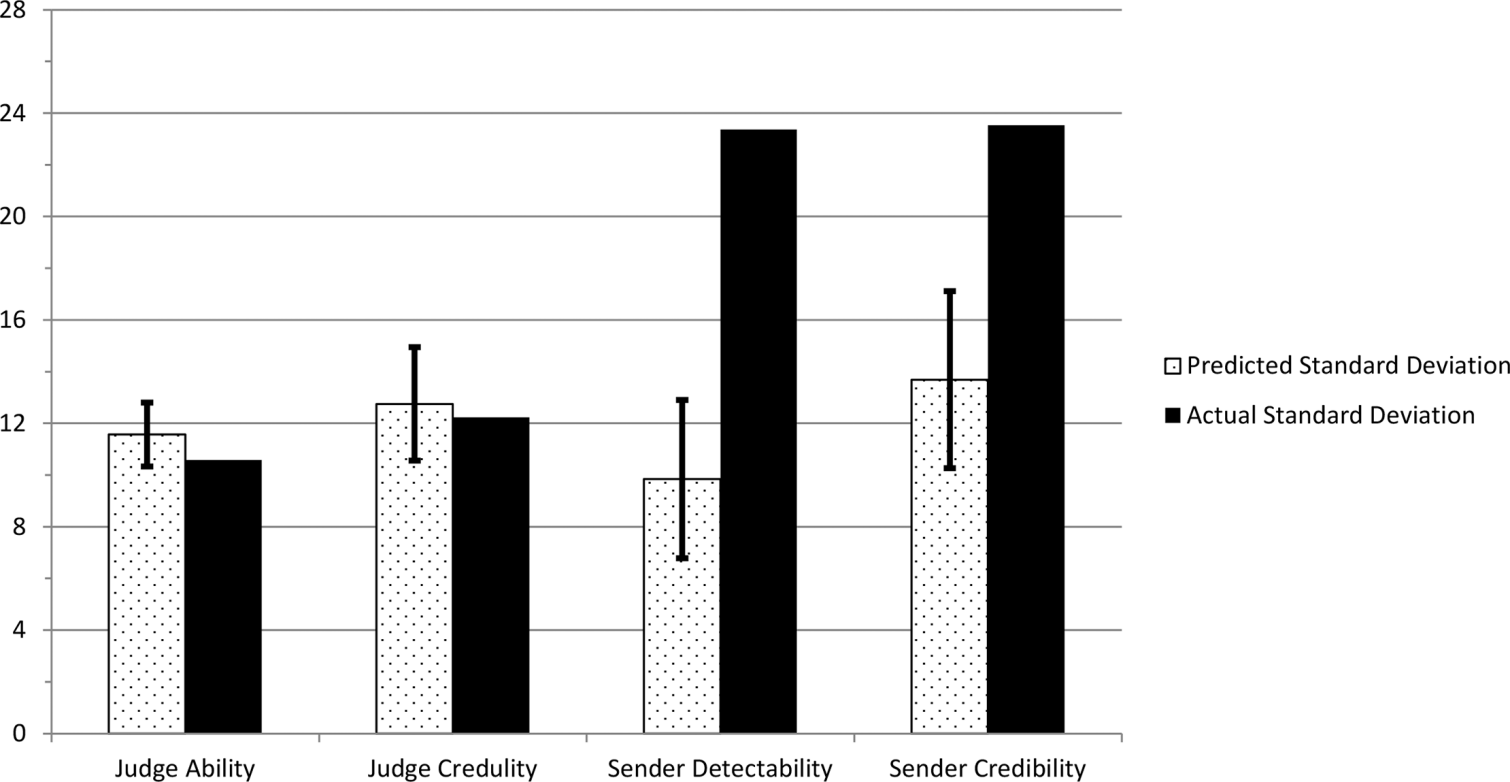
A comparison of the predicted standard deviation from Bond and DePaulo [15] with the actual standard deviations of judge ability, judge credulity, sender detectability and sender credibility in Study 2. Error bars represent the predicted standard deviations within a 95% Confidence Interval.

Using the German sample data, we compared the standard deviations in the four lie detection variables with the predicted standard deviations from Bond and DePaulo [16]’s regression equations (see Fig 2). The predicted values and confidence intervals for each variable were adjusted based on the sample size (117) and the number of sender items (18).

The predicted standard deviations for judge ability, judge credulity, sender detectability and sender credibility were 11.57, 12.76, 9.84 and 13.69 respectively. Given 18 senders and 117 judges, both standard deviations for the judge characteristics fell within the expected 95% confidence intervals. However, the variance of sender characteristics were significantly higher than those predicted by Bond and DePaulo [16]’s regression equations with actual standard deviations of sender detectability and credibility being 23.36 and 23.53 respectively.

#### 3.2.3. Hypothesis 3

No significant correlation was found between judge ability towards senders lying and judge ability towards senders telling the truth within the German sample, (*r* = -.14, *p* > .05).

#### 3.2.4. Hypothesis 4

Only 46 of the 117 participants completed measures of other variables aside from the lie detection task, which gives us insufficient power (1 – β = .54), assuming a two-tailed *p* value (α = .05) to detect even medium size (*r* = .30) correlations. However, similar to Study 1, the lack of support for individual differences in lie detection from the earlier 3 hypotheses suggest that correlations with judge ability would have only been noise.

### 3.3. Discussion

In Study 2, we aimed to examine the hypotheses examined in Bond and DePaulo [16] using a control sample of German native-speakers. The results of all three hypotheses examined in Study 2 supported a lack of individual differences in deception detection. For hypothesis 1, reliability estimates were low for judge characteristics and high for sender characteristics. Examining hypothesis 2, the standard deviations of judge characteristics fell within the predicted values of Bond and DePaulo [16]’s regression equations whilst both standard deviations of the sender characteristics were significantly higher than predicted. Finally, no significant correlation was found between judge ability towards senders lying and senders telling the truth. Although we were not able to examine hypothesis 4 in Study 2, considering the consistency of findings with both Bond and DePaulo [16] and Study 1, it is likely that Hypothesis 4 in Study 2 would have also supported a lack of individual differences in deception detection.

## 4. General discussion

Despite the significant extension of the typical lie-detection paradigm, the two studies presented here provide a consistent replication of previous findings, suggesting that lie detection tasks are ineffective in capturing individual differences in the ability to detect lies. To the best of our knowledge, our Study 1 was the first to extend this conclusion to a lie-detection task in which the truth-telling and lying senders spoke in a foreign language to the receiving judges. These findings add to the evidence suggesting that a focus on judge characteristics is likely to be insufficient for understanding deception detection and predicting its outcomes when para-verbal cues are used. Sender attributes appear to account for greater variance in lie detection rates than do receiving judges’ attributes. The importance of sender characteristics in deception detection may also explain considerable heterogeneity across lie detection studies. Many studies do not properly account for sender characteristics. Thus, the high judging ability scores reported in some studies (e.g., [5]) might have been the result of a task performed by easily detectable senders. Future research could resolve this issue by examining sender characteristics or using standardised lie detection measures when comparing between studies. Furthermore, with increased knowledge about sender characteristics, improved measures might be constructed. By examining relations of item difficulty with sender characteristics and, thus by systematically manipulating these characteristics in item development, measures that actually detect individual differences in deception detection ability might be constructed.

The lack of judge characteristics also demonstrates the importance of strategies that support deception detection within training [58]. Without such strategies, reliable deception detection would be substantially more difficult, if not impossible. We did not find any evidence that lie detection ‘wizards’ exist–at least in our general population samples, and the current study should be replicated with specialist populations such as defence and the police force. If results replicate even in such contexts, training in interrogation techniques would appear to be the only effective method of increasing detection, aligning with existing opinions within the current literature [24, 59].

### 4.1. Limitations and future directions

Despite the convergence with prior research, several limitations of this study must qualify the interpretation of our results. First, the limited number of senders (12 and 18) in the lie detection tasks may have reduced the observed variability. With a greater number of senders, greater variance and less error might be obtained. Still, given the consistency of the results over two studies, it is doubtful that increasing the number of senders will have a drastic effect on the results. Second, the senders in our task were either lying or telling the truth, and only one individual sender did both. Employing the same individuals to present both lies and truthful messages would have been more sensitive to individual differences in sender characteristics—and this method seems worth employing in future studies.

Third, the use of a student and general population sample may have limited the generalisability of our results. Many studies included in Bond and DePaulo’s meta-analysis were also conducted using such samples making our comparisons to previous work valid. However, it also means that generalising our conclusions to specific populations may not be substantiated. Perhaps lie detection is a skill that requires considerable time and experience to develop, making it more likely to emerge in older populations or more targeted populations (e.g., law enforcement officers). Extending future lie-detection studies to such specialised samples would not only improve their generalisability, but also enhance their applicability in practical settings.

### 4.2 Conclusion

Overall, using the novel selection of individual differences measures, the novel research design and the novel statistical approach, our current studies replicated Bond and DePaulo’s findings [15, 16]. Study 1 replicated the findings under the condition of a foreign language-spoken messaging, where the ability of the judging receiver to comprehend the contents of the message is reduced. Removing language comprehension does not appear to reveal stronger individual differences in judge ability in detecting lies. Importantly, identical results were obtained using native German speakers and a longer list of senders in Study 2. Thus, instead of stressing the role of the judging receiver, our findings strongly highlight a role of sender attributes in predicting the outcomes of lie-detecting performance.

## Supporting information

S1 Appendix This document contains Equations A and B. Equation A. This is the equation used within Bond and DePaulo (2008b) to calculate the coefficient alphas for judge and sender characteristics. Equation B. This is Bond and DePaulo (2008a)’s regression equation for predicting the standard deviation of judge and sender characteristics within lie detection tasks.
(DOCX)
