# Supplementary material for: It's the deceiver, not the receiver: No individual differences when detecting deception in a foreign and a native language
Source: PLoS One. 2018 May 3;13(5):e0196384. doi: 10.1371/journal.pone.0196384 (PMC5933718; doi:10.1371/journal.pone.0196384)
Supplement: S1 Appendix — This document contains Equations A and B. Equation A. This is the equation used within Bond and DePaulo (2008b) to calculate the coefficient alphas for judge and sender characteristics. Equation B. This is Bond and DePaulo (2008a)’s regression equation for predicting the standard deviation of judge and sender characteristics within lie detection tasks. (DOCX) [file pone.0196384.s001.docx]

**S1 Appendix**

Formulated equations developed from Bond and DePaulo (2008a, b)

$$\alpha=\left( \frac{n}{n-1} \right) \left( 1-\frac{\sum_{j=1}^{n} pj (1-pj )}{s^{2}x} \right)$$

*Equation. A.* This is the equation used within Bond and DePaulo (2008b) to calculate the coefficient alphas for judge and sender characteristics. For judge ability, ‘α’ is the estimated reliability coefficient, ‘*n*’ is the number of items within the lie detection task, *pj* is the proportion of correct responses on item *j*. *s^2^x* is the variance of the number of items judged correctly across all judges. For a complete report of term meanings, see Table 1 (p. 502) of Bond and DePaulo (2008b).

*S*_i_ = $a +b \left( \frac{1}{\sqrt{ni}} \right)$

*Equation. B.* This is Bond and DePaulo (2008a)’s regression equation for predicting the standard deviation of judge and sender characteristics. ‘*si’* is the predicted standard deviation of the variable for study *i*. ‘*a*’ is the regression intercept, ‘*b*’ is the unstandardized regression coefficient. ‘*ni*’ is the number of ‘senders’ in study *i* when predicting judge characteristics, or number of ‘judges’ in study *i* when predicting sender characteristics.
